# Development of a Digital Sialometry Device for Minor Salivary Glands With Enhanced Volume Range and Precision: A Technical Report

**DOI:** 10.7759/cureus.93385

**Published:** 2025-09-28

**Authors:** Satoshi Gotoh, Hitoshi Kachi, Shizuko Satoh-Kuriwada, Takeo Otabe, Takanori Hatakeyama, Toru Ogawa, Masahiro Iikubo

**Affiliations:** 1 General Dentistry, Haneishi Dental Clinic, Utsunomiya, JPN; 2 Division of Comprehensive Dentistry, Tohoku University Graduate School of Dentistry, Sendai, JPN; 3 Division of Dental Informatics and Radiology, Tohoku University Graduate School of Dentistry, Sendai, JPN; 4 Maxillofacial Prosthetics Clinic, Tohoku University Hospital, Sendai, JPN

**Keywords:** minor salivary gland, oral health care, saliva, saliva testing, sialometry

## Abstract

To address the measurement limitations of a previously developed electronic device for sialometry of minor salivary glands, an improved version was developed to enable accurate assessment of larger saliva volumes. The device estimates saliva volume by calculating the electrical impedance of a filter paper that has absorbed saliva, using a microcontroller-based digital method. A calibration curve was generated using physiological saline, adjusted to match the conductivity of human labial gland saliva. The improved device can measure saliva volumes up to 9.0 μL using a 10 mm square filter paper, significantly expanding the measurable range compared to the previous version. This enhancement increases both the precision and applicability of minor salivary gland sialometry, contributing to a more reliable assessment of salivary function in clinical and research settings.

## Introduction

Saliva plays a fundamental role in maintaining oral health, contributing to lubrication, antimicrobial defense, mucosal protection, and taste perception [1]. It also plays a crucial role in the adhesion and stability of removable dentures, especially in patients with decreased salivary flow [2]. Accurate assessment of salivary gland function is essential in the diagnosis and management of various oral and systemic conditions, including xerostomia, Sjögren’s syndrome, and adverse effects from medications or radiation therapy [3]. In particular, minor salivary glands, which are distributed throughout the oral mucosa, provide valuable diagnostic information because their function can reflect subtle changes in salivary secretion that may not be detected by whole saliva flow rates [4]. Traditional sialometry methods typically measure whole saliva and do not capture site-specific function. By contrast, minor gland sialometry offers a localized assessment that may reveal early or subtle changes. Despite this potential, relatively little clinical research has been conducted on minor gland measurements, largely due to methodological difficulties.

To facilitate quantitative measurement of minor salivary gland secretion, an electronic device was previously developed that estimates saliva volume by measuring the electrical impedance of filter paper after it absorbs saliva [5]. This method enabled non-invasive and site-specific assessment of salivary output. However, the device had a technical limitation in that it could not accurately measure saliva volumes exceeding 3 µL, due to restrictions in its analog impedance measurement circuit.

To address this limitation, the impedance measurement system was redesigned using a digital circuit controlled by a microcontroller. The improved device expands the measurable saliva volume range and enhances the accuracy of impedance-based sialometry, thereby increasing its utility for both clinical and research applications.

## Technical report

**Figure 1:**
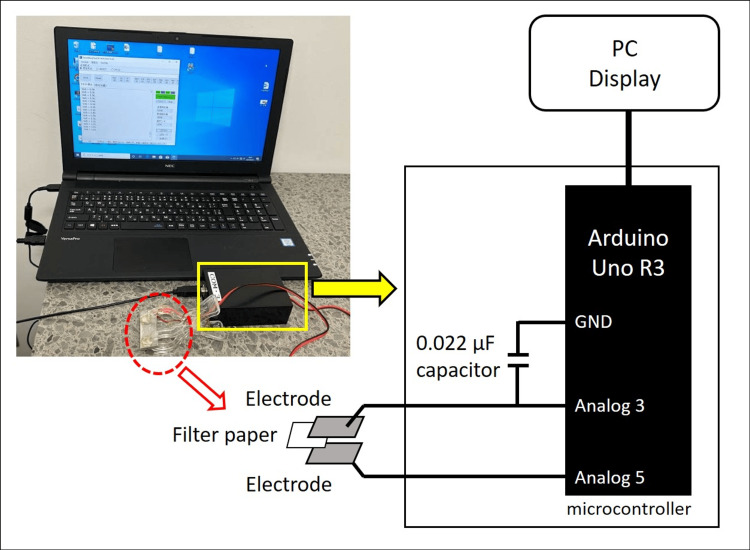
Improved device and its circuit diagram The results are shown on the PC display. Image credit: Photograph courtesy of Dr. Satoshi Gotoh

A digital method was adopted to measure the impedance of the filter paper using a microcontroller (Arduino Uno R3; Arduino LLC, Boston, MA, USA) [6]. In the improved device, a fixed capacitor was connected in series with the filter paper (Figure 1).

The capacitor was charged for a specified duration (ranging from 10 μs to 100 ms), and the microcontroller calculated the impedance based on the charging voltage, capacitance, and charging time. The output value was inversely proportional to the impedance: when the impedance was high, the displayed voltage was low; conversely, when the impedance was low, the displayed voltage was high. As a result, a smaller amount of saliva absorbed by the filter paper yielded a lower output value. The microcontroller program used for impedance calculation was adapted from a digital hygrometer system, using humidity sensors.

The same type of filter paper as that used in our previous study was employed [5]. Specifically, square samples (10 mm × 10 mm) were punched from Filter Paper No. 1 (Toyo Roshi Kaisha, Ltd., Tokyo, Japan), with a standardized thickness of 0.2 mm. The samples were sterilized in an autoclave and stored in sterile containers until use.

To estimate saliva volume, a calibration curve was created based on the measured impedance values. Physiological saline was diluted with purified water and adjusted to match the electrical conductivity of human labial gland saliva, verified using the previously reported device [5]. This method reproduced the calibration values obtained from human saliva and ensured continuity with the previous system.

**Figure 2:**
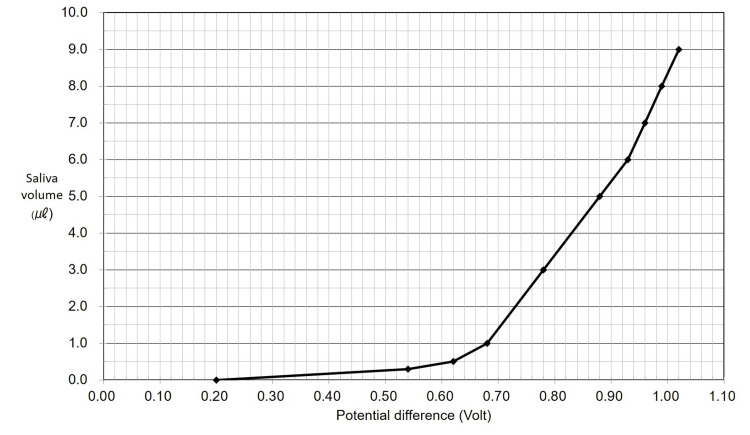
Calibration curve The secretion rate is estimated from a calibration curve.

Quantitative testing using the calibration curve demonstrated the following relationship between saliva volume and potential difference (Figure 2). The corresponding numerical values are presented in Table 1.

**Table 1:** Numerical values of potential difference corresponding to various saliva volumes

Saliva Volume (μL)	Potential Difference (V)
0.0	0.20
0.3	0.54
0.5	0.62
1.0	0.68
3.0	0.78
5.0	0.88
6.0	0.93
7.0	0.96
8.0	0.99
9.0	1.02

Within the linear range from 0.68 V to 0.93 V, the saliva volume (y, in μL) could be calculated using the following regression equation: $y = 20x - 12.6$, where x is the potential difference (in V).

Beyond 9.0 μL, the filter paper reached saturation and could not absorb saliva consistently enough for accurate impedance estimation. Thus, 9.0 μL was determined to be the upper measurable limit of the improved device, approximately three times greater than the maximum measurable volume of the previous version.

## Discussion

In the previously reported device, impedance was measured using an analog method [5]. Specifically, a constant DC voltage was converted into a 1.0 kHz sinusoidal AC signal, which was passed through the filter paper after saliva absorption. The resulting AC signal was log-transformed, rectified, and filtered to obtain a DC signal, which was then measured as a potential difference. A low potential difference indicated high impedance, corresponding to a small amount of absorbed saliva [5]. However, due to limitations in the analog circuitry, the log-transformation function lacked sufficient accuracy in the low-impedance region, resulting in reduced measurement precision.

In contrast, the improved device uses a digital method in which a microcontroller calculates impedance based on capacitor charging behavior. This digital approach maintains accuracy even in the low-impedance range, enabling reliable quantification of saliva volumes up to 9.0 μL, approximately three times greater than the maximum measurable volume of the previous system. This technical enhancement significantly expands the measurable range of minor salivary gland sialometry and increases its potential clinical and research utility.

Another modification involved the calibration procedure. While the previous device used human labial gland saliva for calibration, the improved system employed physiological saline diluted with purified water and adjusted to match the electrical conductivity of labial gland saliva. This adjustment reproduced the calibration values obtained from human saliva, confirming that the new method is both valid and consistent. Therefore, the improved device retains continuity with previous measurements while allowing for greater flexibility in experimental setup.

Limitations and future directions

Saliva Composition

Inter-individual variations in saliva composition were examined in our previous report, where no significant differences were observed among healthy individuals. However, further studies are required in patients with hyposalivation to clarify whether compositional differences impact impedance values.

Viscosity

From an electrical perspective, saliva viscosity is not expected to affect impedance measurements because impedance depends primarily on ionic concentration and conductivity rather than rheological properties.

Filter Paper Saturation

The measurable limit of 9 μL was determined by the saturation capacity of the filter paper, which may constrain higher-volume assessments.

Measurement Protocol

While previous studies have proposed protocols such as 30-second collection with doubled values, we adopted a 60-second collection protocol and used the value directly. Further research is needed to standardize minor salivary gland sialometry protocols.

Clinical Implications

The improved device may facilitate early diagnosis of conditions such as xerostomia, Sjögren’s syndrome, and radiation-induced hyposalivation. Clinical validation studies are required to establish its diagnostic value.

## Conclusions

In this study, we developed an improved digital sialometry device capable of precisely measuring minor salivary gland secretion volumes up to 9.0 μL using a microcontroller-based impedance method. This enhancement expands the measurable range approximately threefold compared to the previous device and greatly improves the reliability of salivary function assessment in both clinical and research settings.
